# Association between Sleep Duration and Cancer Risk: A Meta-Analysis of Prospective Cohort Studies

**DOI:** 10.1371/journal.pone.0074723

**Published:** 2013-09-04

**Authors:** Yan Lu, Nong Tian, Jie Yin, Yuhua Shi, Zhenping Huang

**Affiliations:** Department of Ophthalmology, Jinling Hospital, Nanjing University, Nanjing, China; University College London, United Kingdom

## Abstract

**Background:**

Sleep duration has been shown to play an important role in the development of cancer. However, the results have been inconsistent. A meta-analysis with prospective cohort studies was performed to clarify the association between short or long sleep duration and cancer risk.

**Methods:**

PubMed and Embase databases were searched for eligible publications. Pooled relative risk (RR) with 95% confidence interval (CI) was calculated using random- or fixed- model.

**Results:**

A total of 10 prospective studies (8392 incident cases and 555678 participants) were included in the meta-analysis. Neither short nor long sleep duration was statistically associated with increased risk of cancer (short sleep duration: RR=1.05, 95%CI=0.90-1.24, *p*=0.523; long sleep duration: RR=0.92, 95%CI=0.76-1.12, *p*=0.415). In the subgroup by cancer type, long sleep duration was positively associated with colorectal cancer (RR=1.29, 95%CI=1.09-1.52, *p*=0.003).

**Conclusion:**

The present meta-analysis suggested that neither short nor long sleep duration was significantly associated with risk of cancer, although long sleep duration increased risk of with colorectal cancer. Large-scale well-design prospective studies are required to be conducted to further investigate the observed association.

## Introduction

Cancer is a serious public health problem worldwide. Although many risk factors, including genetic variants, obesity, intake of high-fat food, lack of physical activity, smoking and alcohol consumption, contribute to development of cancer, they can account for only a small proportion of cancer cases. Thus, other unknown risk factors still need to be identified.

Melatonin, synthesized and secreted by the pineal gland in the brain, plays an important role in controlling the body’s circadian rhythm [1]. Evidence has shown that serum melatonin concentrations are lower in habitual short sleepers (<6 h/night) than in long sleepers (>9 h/night) [2]. Thus, sleep duration may influence melatonin levels by determining length of light exposure, thereby affecting cancer risk. Recently, several meta-analyses have indicated that both short and long sleep durations are associated with risk of obesity [3,4], hypertension [5,6], diabetes [7], cardiovascular disease [8], as well as all-cause mortality [9,10]. However, no meta-analysis has been performed to investigate the association between habitual sleep duration and cancer risk, although 10 prospective cohort studies have been conducted and the findings have been inconsistent [11–19]. The discrepancy might be due to the insufficient statistical power of individual study. In this study, therefore, we performed a meta-analysis to clarify the association between sleep duration and cancer risk.

## Materials and Methods

### Literature and search strategy

Literature databases were searched including PubMed and Embase. The search strategy to identify all possible studies involved the use of the following key words: (sleep duration or sleep time) and (cancer or carcinoma). The reference lists of retrieved articles were hand-searched. The literature search was limited to English language. If more than one article were published using the same data, only the study with largest sample size was included. The literature search was updated on May 11, 2013.

### Inclusion criteria and data extraction

The studies included in the meta-analysis met the following inclusion criteria: (1) evaluated the association between sleep duration and cancer risk; (2) used cohort design; (3) provided relative risk (RR) with 95% confidence interval (CI). The following information was extracted from each study: (1) name of the first author; (2) year of publication; (3) origin of country; (4) number of incident cancer cases and total participants; (5) sex ratio and mean age of the study population; (6) duration of follow-up; (7) sleep duration category; (8) cancer type; (9) covariates used in adjustment. The two authors independently assessed the articles for compliance with the inclusion/exclusion criteria and resolved disagreements through discussion.

### Statistical analysis

The association of short or long sleep duration with cancer risk was estimated by calculating pooled RR and 95% CI. The significance of pooled RR was determined by *Z* test (*p*<0.05 was considered statistically significant). A Q test was performed to examine the between-study heterogeneity. A random- (DerSimonian-Laird method [20]) or fixed- (Mantel-Haenszel method [21]) effects model was used to calculate pooled RR in the presence (*p*<=0.10) or absence (*p*>0.10) of heterogeneity, respectively. Subgroup analysis based on cancer type was conducted. Sensitivity analysis after excluding one study at a time was performed to assess the stability of the results. Publication bias was assessed by Begg’s test [22] and Egger’s test [23] (*p*<0.05 was considered statistically significant). Statistical analysis was conducted using STATA version 11 (StataCorp LP, College Station, Texas, USA).

## Results

### Characteristics of the studies

**Figure 1:**
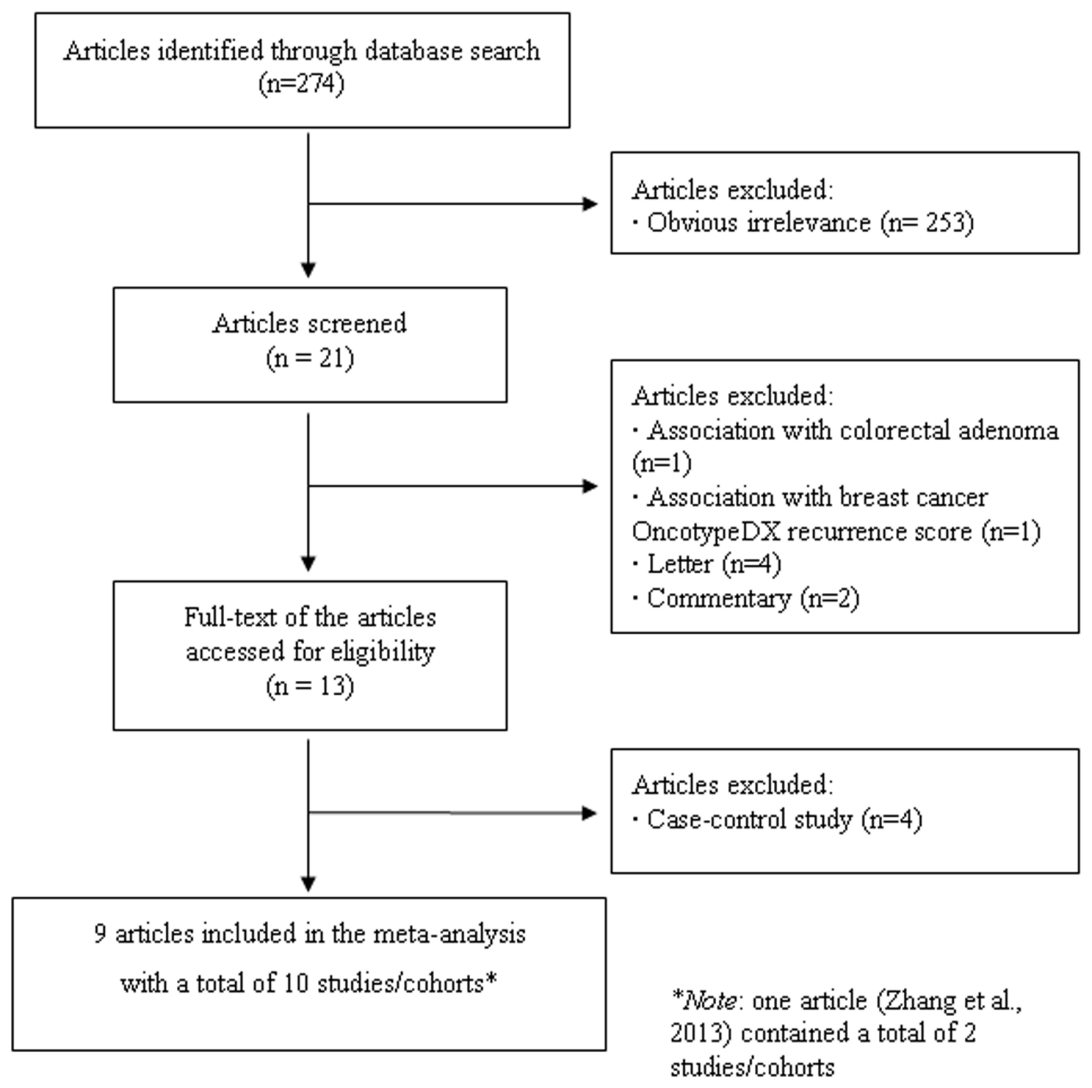
Flow chart of meta-analysis for exclusion/inclusion of individual articles (or studies).

The PRISMA checklist was presented as Table S1. A flow chart for exclusion/inclusion of individual articles (or studies) was presented as Figure 1. The literature search identified a total of 274 potentially relevant papers. 253 papers were excluded after reading the title and abstract because of obvious irrelevance. One paper was excluded since it examined the association between sleep duration and breast cancer OncotypeDX recurrence score. Another paper was excluded because it investigated the association between sleep duration and colorectal adenoma. In addition, four letters and two commentaries were also excluded. Thus, 13 papers met the inclusion criteria. However, four case-control studies were further excluded [24–27]. In addition, since two studies were included in the paper by Zhang et al. [19], they were considered as the separate studies in the following data analysis. Therefore, a total of 9 articles including 10 studies (8392 incident cases and 555678 participants) were included in the final meta-analysis [11–19]. Of them, six studies were on breast cancer, three on colorectal cancer, one on prostate cancer, one on endometrial cancer, one on thyroid cancer, and one on ovarian cancer. Sleep duration category (hours) and other characteristics of the included studies were presented in Table 1.

**Table 1:** Characteristics of the studies included in the meta-analysis.

Study	Country	No. of cases	No. of participants	Sex (male, %)	Age (mean ±SD or age range, years)	Follow-up period (years)	Sleep duration category (hours)	Cancer type	Adjustment
Verkasalo et al, 2005 [11]	Finland	242	11980	All were women	36.5	6	Referent=‘7–8’; Short=‘≤6’; Long=‘≥9’	Breast cancer	Age, zygosity, social class, number of children, use of oral contraceptives, body mass index, alcohol use, smoking, physical activity
Pinheiro et al, 2006 [12]	Austria	4223	73195	All were women	30-50	16	Referent=‘7’; Short=‘≤5’; Long=‘≥9’	Breast cancer	Age, body mass index, height,history of benign breast disease, family history of breast cancer, parity and age at first birth, age at menarche, age at menopause, postmenopausal hormone use, physical activity, smoking.
Kakizaki et al, 2008 [13]	Japan	143	23852	All were women	40–79	9	Referent=‘7’; Short=‘≤6’; Long=‘≥9’	Breast cancer	Age; body mass index; history of diseases; family history of cancer; job; marital status; education; cigarette smoking; alcohol consumption; time spent walking; total caloric intake; menopausal status; age at menarche; age at first delivery; number of deliveries; using of oral contraceptive drugs ; using of hormone drugs except for oral contraceptive drugs
Kakizaki, et al, 2008 [14]	Japan	127	22193	All were men	40–79	9	Referent=‘7–8’; Short=‘≤6’; Long=‘≥9’	Prostate cancer	Age; marital status; education; job status; history of diseases; family history of cancer ; body mass index; cigarette smoking; alcohol consumption; walking status
Sturgeon et al, 2012 [15]	USA	452	48273	All were women	50–79	7.5	Referent=‘7’; Short=‘≤6’; Long=‘≥9’	Endometrial cancer	Age, race, body mass index, smoking, number of live births, physical activity, unopposed estrogen use, oral contraceptive use, and family history of endometrial cancer
Luo et al, 2012 [16]	USA	295	142638	All were women	50–79	11	Referent=‘7–8’; Short=‘≤6’; Long=‘≥9’	Thyroid Cancer	Age at enrollment, ethnicity, educational level, smoking, BMI, recreational physical activity, alcohol intake, family history of cancer, previous thyroid disease, history of hormone therapy use, depression score, and different treatment assignments for Women’s Health Initiative clinical trials
Weiderpass et al, 2012 [17]	Japan	86	45662	All were women	40–69	7.6	Referent=‘6–7’; Short=‘<6’; Long=‘>7’	Ovarian cancer	Age, study center, age at menarche, nulliparous, parity, age at first birth, breastfeeding, use of exogenous hormones, menopausal status at enrollment, height, body mass index, smoking status, exposure to second-hand smoke, alcohol consumption, physical activity, family history of cancer
Jiao et al, 2013 [18]	USA	851	74977	All were women	63±7	11.3	Referent=‘7’; Short=‘≤5’; Long=‘≥9’	Colorectal cancer	Age, ethnicity, fatigue, hormone replacement therapy, waist to hip ratio, and physical activity
Zhang et al, 2013 [19]	USA	709	29412	All were men	41-79	22	Referent=‘7’; Short=‘≤5’; Long=‘≥9’	Colorectal cancer	Age, smoking before age 30, history of colorectal cancer in a parent or sibling, history of endoscopy, regular aspirin use, physical activity, snoring, body mass index, history of diabetes, beef, pork, and lamb as a main dish, consumption of processed meat, alcohol consumption, energy-adjusted total calcium intake, total folate, and total vitamin D intake
Zhang et al, 2013 [19]	USA	1264	75104	All were women	40-73	22	Referent=‘7’; Short=‘≤5’; Long=‘≥9’	Colorectal cancer	Age, smoking before age 30, history of colorectal cancer in a parent or sibling, history of endoscopy, regular aspirin use, physical activity, snoring, body mass index, history of diabetes, beef, pork, and lamb as a main dish, consumption of processed meat, alcohol consumption, energy-adjusted total calcium intake, total folate, total vitamin D intake, postmenopausal hormone use

### Meta-analysis results

**Figure 2:**
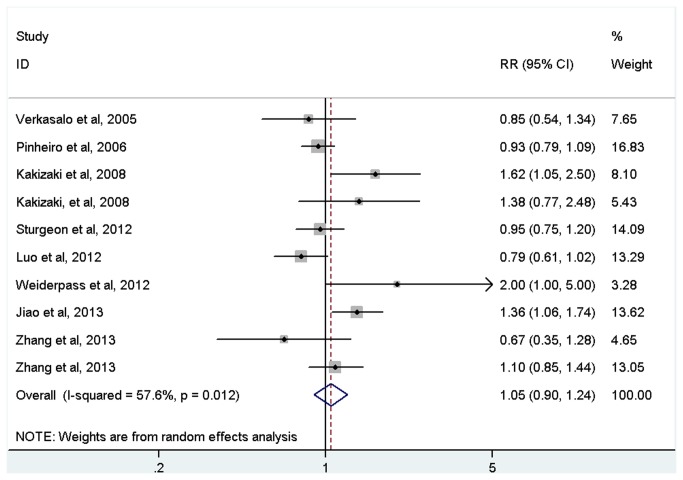
Meta-analysis of the association between short sleep and risk of cancer.

Short sleep duration was not statistically associated with increased risk of cancer (RR=1.05, 95%CI=0.90-1.24, *p*=0.523; Figure 2 and Table 2) with evidence of between-study heterogeneity (*I* ^2^=57.6%, *p* for heterogeneity=0.012). In the subgroup by cancer type, non-significant association was also found for all cancers (Table 2).

**Table 2:** Meta-analysis of the association between sleep duration and cancer risk.

Group	No. of studies (incident cases)	RR	95%CI	P z	Statistical model	*I* ^2^ (%)	*P*_H_
Short sleep							
All	10 (8392)	1.05	0.90-1.24	0.523	Random	57.6	0.012
Cancer type							
Breast cancer	3 (4608)	1.06	0.75-1.49	0.739	Random	66.3	0.051
Colorectal cancer	3 (2824)	1.12	0.84-1.49	0.449	Fixed	55.2	0.107
Prostate cancer	1 (127)	1.38	0.77-2.48	0.280	-	-	-
Endometrial cancer	1 (452)	0.95	0.75-1.20	0.669	-	-	-
Thyroid cancer	1 (295)	0.79	0.61-1.02	0.072	-	-	-
Ovarian cancer	1 (86)	2.00	0.89-4.47	0.091	-	-	-
Long sleep							
All	10 (8392)	0.92	0.76-1.12	0.415	Random	68.9	0.001
Cancer type							
Breast cancer	3 (4608)	0.88	0.73-1.07	0.192	Fixed	15.1	0.308
Colorectal cancer	3 (2824)	1.29	1.09-1.52	0.003	Fixed	5.8	0.346
Prostate cancer	1 (127)	0.36	0.18-0.72	0.004	-	-	-
Endometrial cancer	1 (452)	0.83	0.49-1.40	0.487	-	-	-
Thyroid cancer	1 (295)	0.74	0.39-1.40	0.353	-	-	-
Ovarian cancer	1 (86)	0.80	0.65-0.99	0.038	-	-	-

Abbreviations: OR, odds ratio; CI, confidence interval

P z, *P* value for Z test. *P*_H_, *P* value based on Q test for between-study heterogeneity

**Figure 3:**
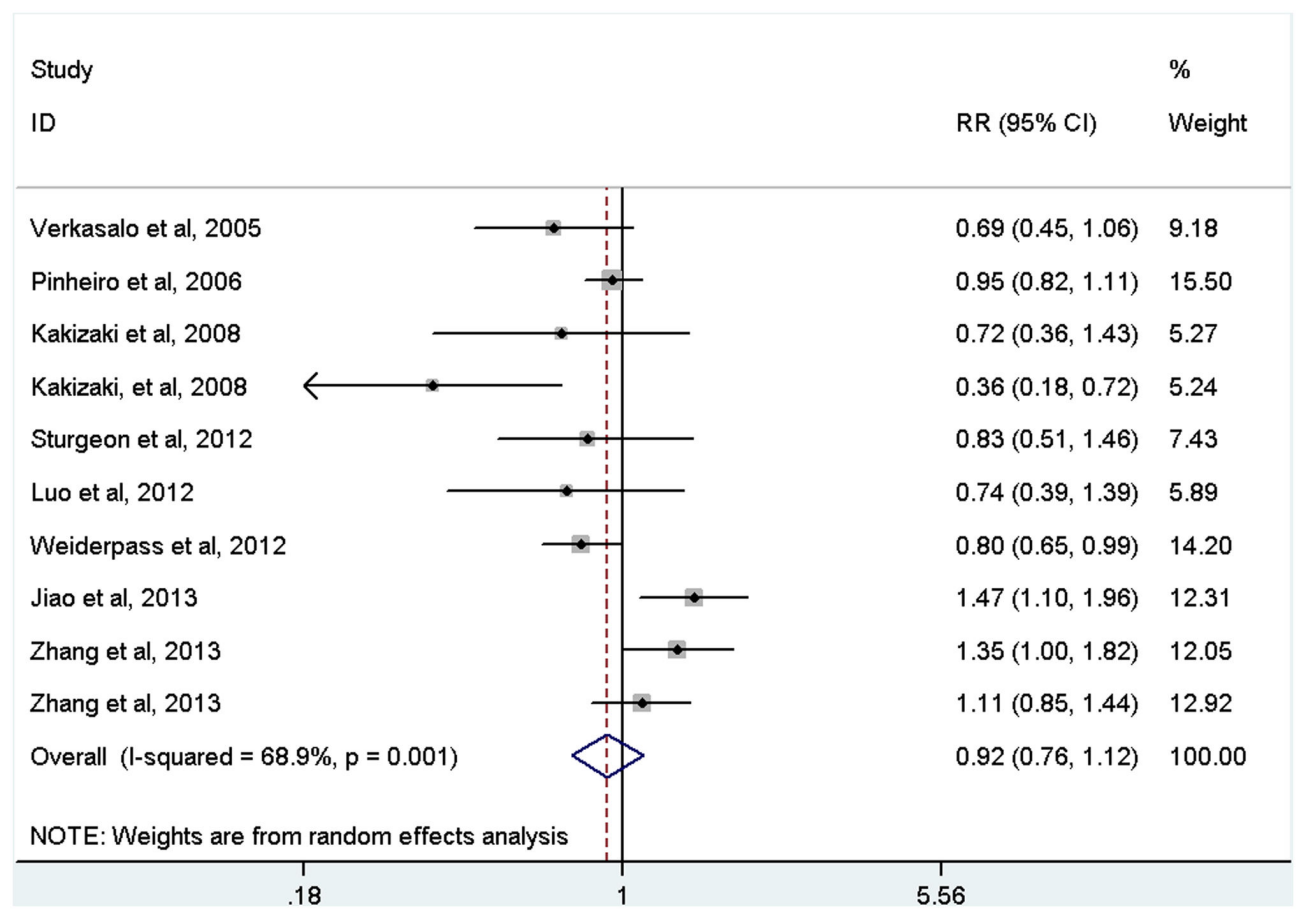
Meta-analysis of the association between long sleep and risk of cancer.

Long sleep duration was not statistically associated with increased risk of cancer (RR=0.92, 95%CI=0.76-1.12, *p*=0.415; Figure 3 and Table 2) with evidence of between-study heterogeneity (*I* ^2^=68.9%, *p* for heterogeneity=0.001). In the subgroup by cancer type, long sleep duration was positively associated with colorectal cancer (RR=1.29, 95%CI=1.09-1.52, *p*=0.003), but inversely associated with prostate cancer (RR=0.36, 95%CI=0.18-0.72, *p*=0.004) and ovarian cancer (RR=0.80, 95%CI=0.65-0.99, *p*=0.038). There was no significant association of long sleep duration with endometrial cancer and thyroid cancer (Table 2).

### Sensitivity analysis and publication bias

After excluding one study at a time, the sensitivity analysis confirmed the non-significant association between sleep duration and cancer risk (data not shown). No publication bias was detected for short sleep duration (Begg’s test: *p*= 0.592 and Egger’s test: *p*= 0.349) and long sleep duration (Begg’s test: *p*= 0.210 and Egger’s test: *p*= 0.374).

## Discussion

To the best of our knowledge, this is the first meta-analysis with prospective cohort studies examining the association between sleep duration and cancer risk. The findings showed that neither short nor long sleep duration was significantly associated with cancer risk. In the subgroup analysis by cancer type, we found long sleep duration was positively associated with colorectal cancer, but inversely associated with prostate cancer and ovarian cancer.

To date, six studies have investigated the association between sleep duration and breast cancer and have revealed mixed results [11–13,25–27]. Three studies were cohort based [11–13] and the other three studies were case-control based [25–27]. In this meta-analysis, we did not recruit three case-control studies since they limit casual inference. The pooling of the RRs from three cohort studies did not suggest any association for either short or long sleep duration with breast cancer. In the subgroup analysis by cancer type, we observed positive association between long sleep duration and colorectal cancer risk. This finding was based on three cohort studies including 2824 incident cases. We think the result was credible since sufficient statistical power was achieved. Regarding to the inverse association of long sleep duration with prostate cancer and ovarian cancer, we believe the result might be due to chance since only one study with small cases was included for each cancer. Thus, further cohort studies with sufficient statistical power are required to confirm or refute the findings.

In this meta-analysis, about 8,392 incident cases and 555,678 participants were included, thus, we have sufficient statistical power to draw the conclusion. In addition, all included studies were prospective cohort based, thus, the conclusion is more convincing. Also, covariates’ estimates from individual study were used to pool the results. Thus, the estimate is more exact and the conclusion is more credible. However, our study is subject to four limitations. First, all included studies measured the sleep duration using the subjective questionnaire rather than the objective actigraphy. However, self-reported sleep duration assessment is well correlated with values obtained by actigraphic monitoring [28]. Second, the confounding variables controlled for were different between studies. Further well-designed studies with consideration of more covariates are required to examine the association between sleep duration and cancer risk. Third, we had no data on sleep quality, the presence of sleeping disorders or rotating shift work, which may influence sleep duration and the association with cancer risk. Fourth, there was significant heterogeneity between studies. Thus, meta-regression meta-analysis was performed to examine source of heterogeneity, with introduction of variables including type of cancers, number of cases, mean age of subjects and duration of follow-up. The results suggested that type of cancers was the main source of heterogeneity (*p*<0.05). In the subgroup analysis by type of cancers, the heterogeneity disappeared in each subgroup with the exception for the subgroup of the association between breast cancer risk and short sleep duration.

In summary, the present meta-analysis suggested that neither short nor long sleep duration was significantly associated with risk of cancer, although long sleep duration increased risk of with colorectal cancer. Large-scale well-design prospective studies are necessary to be conducted to further confirm or refute the observed association.

## Supporting Information

Table S1 **PRISMA Checklist.**
(DOC)
